# Movable and Focus-Tunable Lens Based on Electrically Controllable Liquid: A Lattice Boltzmann Study

**DOI:** 10.3390/e24121714

**Published:** 2022-11-24

**Authors:** Fei Wang, Zijian Zhuang, Zhangrong Qin, Binghai Wen

**Affiliations:** 1 Guangxi Key Lab of Multi-Source Information Mining & Security, Guangxi Normal University, Guilin 541004, China; 2 School of Computer Science and Engineering, Guangxi Normal University, Guilin 541004, China

**Keywords:** liquid lens, electrically controllable liquid, electrowetting-on-dielectrics, lattice Boltzmann method

## Abstract

Adjusting the focal length by changing the liquid interface of the liquid lens has become a potential method. In this paper, the lattice-Boltzmann-electrodynamic (LB-ED) method is used to numerically investigate the zooming process of a movable and focus-tunable electrowetting-on-dielectrics (EWOD) liquid lens by combining the LBM chemical potential model and the electrodynamic model. The LB method is used to solve the Navier–Stokes equation, and the Poisson–Boltzmann (PB) equation is introduced to solve the electric field distribution. The experimental results are consistent with the theoretical results of the Lippmann–Young equation. Through the simulation of a liquid lens zoom driven by EWOD, it is found that the lens changes from a convex lens to a concave lens with the voltage increases. The focal length change rate in the convex lens stage gradually increases with voltage. In the concave lens stage, the focal length change rate is opposite to that in the convex lens stage. During the zooming process, the low-viscosity liquid exhibits oscillation, and the high-viscosity liquid appears as overdamping. Additionally, methods were proposed to accelerate lens stabilization at low and high viscosities, achieving speed improvements of about 30% and 50%, respectively. Simulations of lens motion at different viscosities demonstrate that higher-viscosity liquids require higher voltages to achieve the same movement speed.

## 1. Introduction

The adaptive zoom lens is essential in photography, projection, medicine, machine vision, augmented reality, virtual reality, and other fields [1,2,3,4,5,6,7,8,9]. The liquid lens can achieve adjustable focal length by changing the liquid interface, overcoming the disadvantages of mechanical movable parts, complex structure, being easy to wear, and difficult miniaturization of the traditional lens, and thus is favored by researchers. Up to now, the liquid lens can be roughly divided into three categories: (1) mechanical force liquid lens [10,11,12], which uses a micropump to squeeze another liquid container connected to the liquid lens to change the curvature of the lens; (2) dielectric force liquid lens [13,14,15], where the focal length is changed by applying an external voltage to make the lens change its shape under the action of a non-uniform gradient electric field; and (3) Electrowetting-on-dielectrics (EWOD) liquid lens [16,17,18,19], where the focal length is adjusted by changing the curvature of the liquid interface, which can be controlled by adjusting the surface tension between solid and liquid through applying an external voltage. When the voltage is applied, the three-phase contact line will generate an outward thrust, the contact angle will decrease, and the curvature will be reduced. In these technologies, the electrowetting liquid lens can achieve continuous zoom without mechanical movement and has the advantages of low cost, small size, high optical performance, simple structure, robust functionality, etc., and thus has a broad development prospect. At the earliest, Berger et al. and Kuiper et al. [1,20] independently produced and industrialized the liquid lens based on electric wetting technology. Choi et al. [21] designed a new electrically focus-tunable double convex lens composed of an nPVC gel and two plates with electrodes, without any stretchable or transparent electrodes and additional actuation mechanisms. Song et al. [22] proposed an electrowetting-based triple-liquid lens whose numerical aperture is larger than the traditional electrowetting lens. Recently, Xu et al. [2] presented an adaptive liquid lens using a novel transparent electrically responsive fluid, dibutyl adipate. The liquid lens with a hemispherical plano-convex shape can change its curvature according to the application of various input voltages. The current work done by previous authors on liquid lens provides a good basis for an in-depth study of the theory and application of liquid lenses. However, there is still progress to be explored in terms of enhancing the zoom speed and driving the lens movement.

The method used in this study is the chemical potential multiphase model proposed by Wen et al. [23]. Based on the mesoscopic scale, the lattice Boltzmann model (LBM) can deal with complex interface deformation without interface tracking. The model has a great density ratio, Galilean invariance, and thermodynamic consistency. An optional proportional coefficient is introduced to decouple the computational mesh from the momentum space [23]. Together with a high-precision compact finite-difference format, the ordinary EOS can achieve extremely large density ratios, while the thermodynamic consistency is still preserved and the spurious current is suppressed to a very low level.

In this paper, the lattice-Boltzmann-electrodynamic (LB-ED) method is used to study the zooming process of a liquid lens by combining the lattice-Boltzmann method chemical potential model and the electrodynamic model. The LB method is used to solve the Navier–Stokes equation, and the Poisson–Boltzmann (PB) equation is introduced to solve the electric field distribution, and the five-point difference is applied to calculate the electric force that drives the zoom of the lens. In Section 2, we briefly introduce the principle of the electrically wetted lens, and the model used in this paper. In Section 3, we simulate the static equilibrium contact angle of the lens under the action of an applied voltage. The experimental results were compared with the well-known Lippmann–Young equation under different wall wetting conditions to verify the accuracy of the model. In Section 4, the relationship between voltage and focal length is simulated by different voltage. By simulating and analyzing the effects of liquid media with different viscosities on the lens performance, methods are proposed to accelerate the stable imaging of the lens for high and low liquid concentrations. By simulating the process of the electrode array driving the lens motion, we found that the high viscosity has a relatively large resistance to the lens motion. In Section 5, we conclude this paper with a brief summary of our work.

## 2. Theoretical Method and Numerical Model

### 2.1. Schematic and Principle

**Figure 1:**
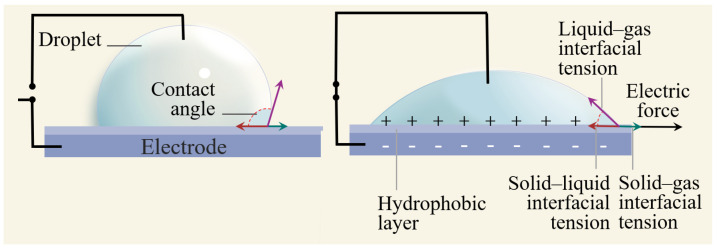
EWOD schematic diagram.

In order to better understand the electric wetting lens, we first briefly introduce the principle of electric wetting. As shown in Figure 1, electrolysis can be effectively avoided by coating a dielectric layer on the metal electrode. The force balance determines the contact angle at the three-phase contact point. When the pressure and temperature are determined, the equilibrium contact angle can be expressed by Young’s equation [24]:

(1) $$\cos\theta =\frac{\gamma_{sv}-\gamma_{sl}}{\gamma_{lv}},$$

where $\gamma_{sv}$, $\gamma_{sl}$, and $\gamma_{lv}$ are the surface tensions of solid–gas, solid–liquid, and liquid–gas interfaces, respectively.

When voltage is applied, the wettability changes, and the equilibrium contact angle of droplets gradually decreases. It was initially discovered by Lippmann’s study of the electric capillary phenomenon, where a capillary rise of the liquid occurs when a voltage is applied between the mercury liquid and the electrolyte [25]. The accumulation of electric charge changes the solid–liquid interfacial tension, which can be expressed as

(2) $$\sigma_{e}=-\frac{\partial \gamma_{sl}}{\partial V}.$$

(3) $$c_{H}=\frac{\partial \sigma_{e}}{\partial V},$$

where $\sigma_{e}\;(c/m^{2})$ is the surface charge density, V(V) is the applied voltage, and $c_{H}\;(F/m^{2})$ is the capacitance per unit area of the electric double layer. Based on Equations (2) and (3), we can obtain the relationship between voltage and surface tension at the solid–liquid interface [26]:

(4) $$\gamma_{sl}=\gamma_{sl0}-\frac{1}{2}C_{H}V^{2},$$

where $\gamma_{sl}$ and $\gamma_{sl0}$ are the solid–liquid interfacial tension with and without voltage applied, respectively. The static equilibrium contact angle in the initial state is

(5) $$\cos\theta_{0}=\frac{\gamma_{sv}-\gamma_{sl0}}{\gamma_{lv}}.$$

Substitute Equations (4) and (5) into Equation (1); after some reformatting, the equilibrium contact angle can be expressed as [26,27]:

(6) $$\cos\theta =\cos\theta_{0}+\frac{\varepsilon_{0}\varepsilon_{r}}{2d\gamma_{lv}}V^{2},$$

where θ is the static contact angle at the supplied voltage *V*, *d* is the thickness of the dielectric layer, $\varepsilon_{0}$ is the vacuum permittivity, and $\varepsilon_{r}$ is the liquid medium’s relative permittivity. Among them, the last term is the electrowetting constant term:

(7) $$\eta =\frac{\varepsilon_{0}\varepsilon_{r}}{2d\gamma_{lv}}V^{2}.$$

**Figure 2:**
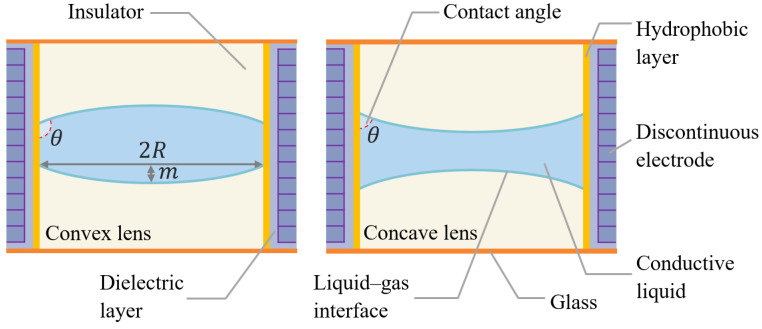
Schematic diagram of the electrowetting lens structure.

Figure 2 is a schematic structural diagram of a variable focus liquid lens. A discontinuous electrode and a dielectric layer coated with a hydrophobic substance are attached to the inner wall of the lens cavity. Fill the lens cavity with two substances (one is a conductor, and the other is an insulator). The contact angle between the conductive liquid and the inner wall can be reduced by applying a voltage. As the curvature of the interface decreases, the angle of light incidence decreases, thus increasing the lens’s focal length. By controlling the state of the discontinuous electrodes, the moving zoom of the lens can be realized [28].

### 2.2. LB-ED Numerical Method

#### 2.2.1. The Multiple-Relaxation-Time LBM

The lattice Boltzmann method [29], which originated from cellular automata and the kinetic theory of molecules, has been developed into a promising numerical method for fluid mechanics, which has an excellent performance in turbulent flow, multiphase flow, microfluid, thermal flow, and other fields. LBM facilitates the handling of complex geometric boundary conditions without the need to trace the interface [30,31,32]. Since the collision of lattice points is only related to the current node, the LBM method is particularly suitable for massive parallelism [33]. With the development of GPUs, the efficiency of simulation using the LBM has been tremendously improved.

In this paper, the multiple relaxation time Boltzmann evolution equation is used [34,35,36,37]

(8) $$f_{i}(x+e_{i}\delta t,t+\delta t)-f_{i}(x,t)=-M^{-1}\cdot S\cdot \left[m-m^{\left(eq\right)}\right]+F_{i},$$

where $f_{i}\left(x,t\right)$ is the distribution function of the particle at the lattice site x and time *t*, and δt is the time interval. The discrete lattice speeds $e_{i}$ define the moving direction, S is a diagonal matrix containing multiple relaxation times, denoted as $S\;=\mathrm{diag}(s_{\rho },s_{\sigma },s_{\varsigma },s_{j},s_{q},s_{j},s_{q},s_{\upsilon },s_{\upsilon })$ [38], M is a linear transformation matrix, which implements the interconversion between the distribution function space and the moment space, m=M·f and $m^{\left(eq\right)}=M\cdot f^{\left(eq\right)}$ represent the velocity moment and the corresponding equilibrium moment of the particle distribution function, respectively. In this study, the relaxation time is taken as $s_{\rho }=s_{j}=1$, $s_{\sigma }=1.64$, $s_{\varsigma }=1.54$, $s_{q}=1.7$ and $s_{\upsilon }=1/\tau$ [38], where τ is the dimensionless relaxation time. The external force term $F_{i}$ uses the exact difference proposed by Kupershtokh et al. [39], which is expressed as the difference of a time-step equilibrium distribution function:

(9) $$F_{i}={f_{i}}^{\left(eq\right)}(\rho ,u+\delta u)-{f_{i}}^{\left(eq\right)}(\rho ,u),$$

$f_{i}^{\left(eq\right)}(x,t)$ is the equilibrium distribution function, and the expression is [40]

(10) $$f_{i}^{\left(eq\right)}(x,t)=\omega_{i}\rho \left[1+\frac{\left(e_{i}\cdot u\right)}{c_{s}^{2}}+\frac{{\left(e_{i}\cdot u\right)}^{2}}{c_{s}^{2}}-\frac{u^{2}}{2c_{s}^{2}}\right],$$

where $\omega_{i}$ is the weight coefficient of particles in different directions, $c_{s}=c/\sqrt{3}$ is the speed of sound of the lattice, ρ and u are the macroscopic density and the macroscopic velocity, respectively. In this paper, the D2Q9 model is used, and the following formulas give the discrete speed and weight of each direction:

(11) ei=010−101−1−110010−111−1−1,ωi=449,i=09,i=0119,i=1∼49,i=1∼41/36,i=5∼8.

#### 2.2.2. Multiphase Model Based on Chemical-Potential

In this paper, the chemical potential lattice Boltzmann method for multiphase flow is employed [41,42,43,44,45,46]. In van der Waals (VDW) fluids, the free energy generalization containing the gradient squared approximation can be expressed as [38,47,48,49]

(12) $$\Psi =\int \left[\psi \left(\rho \right)+\frac{\kappa }{2}{\left|\nabla \rho \right|}^{2}\right]dx,$$

where the first term on the right-hand side of the equation is the free energy density at a temperature of *T*, and the second term is the contribution of the density gradient to the free energy in a non-uniform system, κ is the surface tension coefficient, and ρ is the density. The calculation of the chemical potential can be based on the density and the free energy density:

(13) $$\mu =\psi^{\prime }\left(\rho \right)-\kappa \nabla^{2}\rho .$$

The free energy function determines the diagonal term of the pressure tensor:

(14) $$p=p_{0}-\kappa \rho \nabla^{2}\rho -\frac{\kappa }{2}{\left|\nabla \rho \right|}^{2},$$

where the general expression for the fluid state equation is

(15) $$p_{0}=\rho \psi^{\prime }\left(\rho \right)-\psi \left(\rho \right).$$

The well-known Peng–Robinson (PR) EOS is superior in expressing the density of the liquid phase [50]:

(16) $$p_{0}=\frac{\rho RT}{1-b\rho }-\frac{\alpha \alpha \left(T\right)\rho^{2}}{1+2b\rho -b^{2}\rho^{2}},$$

and its chemical potential is

(17) $$\mu_{}^{\mathrm{PR}}=RT\ln\frac{\rho }{1-b\rho }-\frac{a\alpha (T)}{2\sqrt{2}b}\ln\frac{\sqrt{2}-1+b\rho }{\sqrt{2}+1-b\rho }+\frac{RT}{1-b\rho }-\frac{a\alpha (T)\rho }{1+2b\rho -b^{2}\rho^{2}}-\kappa \nabla^{2}\rho ,$$

where *R* is the gas constant, *a* is the attraction parameter, *b* is the volume correction parameter, and the temperature function is ${\left[1+\left(0.37464+1.54226\omega -0.26992\omega^{2}\right)\left(1-\sqrt{T/T_{c}}\right)\right]}^{2}$. In this paper, the parameters are given by a=2/49, b=2/21, and R=1. The acentric factor ω is 0.344 for water. To make the numerical results match better with the actual physical properties, we define the reduced variables $T_{r}=T/T_{c}$ and $\rho_{r}=\rho /\rho_{c}$, in which $T_{c}$ is the critical temperature, and $\rho_{c}$ is the critical density. To improve the stability of the multiphase flow system at large density ratios, a proportional coefficient *k* is introduced to decouple the dimension units of the length between the momentum space and the lattice space, namely $\delta \hat{x}=k\delta x$. Superscripts are used here to distinguish quantities in mesh space. The chemical potential in mesh space can be transformed into the following formula [23,43]:

(18) $$\hat{\mu }=k^{2}\psi^{\prime }\left(\rho \right)-\hat{\kappa }\;{\hat{\nabla }}^{2}\rho \;.$$

This mesh transformation is mathematically equivalent without accuracy loss. The thermodynamic pressure tensor can be written as

(19) $$P_{\alpha \beta }=p\delta_{\alpha \beta }+\kappa \frac{\partial \rho }{\partial x_{\alpha }}\frac{\partial \rho }{\partial x_{\beta }}.$$

where $\delta_{\alpha \beta }$ is the Kronecker delta function. By substituting Equation (13) into Equation (19), the relationship between the divergence of the pressure tensor and the chemical potential gradient can be obtained after a series of transformations:

(20) ∇·P=ρ∇μ.

At ideal gas pressure, the nonideal force can be calculated based on the overpressure part [23,42,43,44,51]:

(21) $$F=-\nabla \cdot P+\nabla \cdot P_{0},$$

From Equations (20) and (21), it is easy to obtain the formula for calculating the nonideal force based on the chemical potential, which contains only the density and the free energy density:

(22) $$F=-\rho \nabla \mu +c_{S}^{2}\nabla \rho .$$

#### 2.2.3. Electrodynamic Model

The Poisson–Boltzmann (PB) equation is a differential equation used to calculate the ion concentration and charge density distribution in electrolyte solutions. Before calculating the electric field forces acting on the lens, we can use the PB equation to solve the potential distribution.

The Boltzmann distribution can describe the movement of free charges in solution. The Boltzmann equation is used to count the ion density of *i*, given as

(23) $$c_{i}=c_{i}^{0}\cdot \exp\left(\frac{-W_{i}}{k_{B}T}\right),$$

where $c_{i}^{0}$ is the bulk concentration of the ion, $k_{B}$ is the Boltzmann constant ($k_{B}=1.381\times 10^{-23}\;m^{2}\cdot k^{-1}\cdot s^{-2}$ ), *T* is the absolute temperature, and $W_{i}$ is the average force potential energy of the ion *i*. Assume that the work done by the ions is only electrical work. For an electrolyte with chemical valence 1:1, the work required to bring a positively or negatively charged ion to a surface with potential φ can be expressed as $W^{+}=e\varphi$ and $W^{-}=-e\varphi$, respectively. The local charge density can be written as [52,53]

(24) $$\rho_{e}=e\left(c^{+}-c^{-}\right)=c_{0}e\cdot \left[\exp\left(\frac{-e\varphi }{k_{B}T}\right)-\exp\left(\frac{e\varphi }{k_{B}T}\right)\right],$$

where *e* is the elementary charge ($e=1.6\times 10^{-19}\;C$). Another basic equation is the Poisson equation, which calculates the electrochemical potential of ions in the diffusion layer. The second partial derivative of the PB equation is [52]

(25) $$\frac{d^{2}\varphi }{dx^{2}}=-\frac{\rho_{e}}{\varepsilon_{0}\varepsilon_{r}}=\frac{c_{0}e}{\varepsilon_{0}\varepsilon_{r}}\cdot \left[\exp\left(\frac{e\varphi }{k_{B}T}\right)-\exp\left(\frac{-e\varphi }{k_{B}T}\right)\right].$$

where $\varepsilon_{0}$ is the dielectric constant in vacuum ($\varepsilon_{0}=8.85\times 10^{-12}\;C^{2}\cdot J^{-1}\cdot m^{-1}$), $\varepsilon_{r}$ is the relative dielectric constant ($\varepsilon_{r}=81$), $\rho_{e}$ is the charge density per unit volume. In dilute solution, the absolute value of the potential energy of the ion is small ($e\varphi /k_{B}T\ll 1$), and the exponential term in the PB equation can be expanded to the first order as

(26) $$\frac{d^{2}\varphi }{dx^{2}}=\frac{2c_{0}e^{2}\varphi }{\varepsilon_{0}\varepsilon_{r}k_{B}T}=k^{2}\varphi ,$$

where $k^{-1}$ is the length of Debye, which is only related to the electrochemical properties of the solution. In the two-dimensional Cartesian coordinate system, taking δx=δy, the electric potential can be expressed as

(27) $$\varphi_{i,j}=\frac{2\left(\varphi_{i,j+1}+\varphi_{i,j-1}+\varphi_{i+1,j}+\varphi_{i-1,j}\right)+\left(\varphi_{i+1,j+1}+\varphi_{i+1,j-1}+\varphi_{i-1,j+1}+\varphi_{i-1,j-1}\right)}{3\left(k^{2}\delta x^{2}+4\right)}.$$

Since the relative permittivity of the conducting part is significantly larger than that of the insulating part (water vapor in this paper), the ionic density in the air can be neglected. The boundary conditions at the gas–liquid interface can be expressed as [54]:

(28) n·∇φ=0,

where n is the unit normal vector at the gas–liquid interface.

Because there is no free charge in the gas phase region, the potential satisfies the Laplace equation [55]:

(29) $$\nabla^{2}\varphi =\frac{\partial^{2}\varphi }{\partial x^{2}}+\frac{\partial^{2}\varphi }{\partial y^{2}}=0.$$

The permittivity ε can be expressed as [56]

(30) $$\varepsilon \left(\rho \right)=\frac{\rho -\rho_{l}}{\rho_{v}-\rho_{l}}\varepsilon_{v}-\frac{\rho -\rho_{v}}{\rho_{v}-\rho_{l}}\varepsilon_{l},$$

where $\varepsilon_{l}$ and $\varepsilon_{v}$ are the dielectric constants of the liquid and gas phases, respectively. $\rho_{l}$ and $\rho_{v}$ are the densities of the liquid and gas phases, respectively.

The field strength at any point in the electric field is equal to the negative value of the potential gradient at that point, namely E=−∇φ. Combining Equation (25), we can solve the electric field force [57]:

(31) $$F_{e}=\rho_{e}E-\frac{1}{2}{\left|E\right|}^{2}\nabla \varepsilon +\nabla \left[\rho \frac{{\left|E\right|}^{2}}{2}\frac{\partial \varepsilon }{\partial \rho }\right],$$

where the first term is the response of the free charge density ρ in the system; the second term on the right side is the polarization stress; and the third term is that the electrostriction force is negligible for incompressible fluids.

## 3. Model Verification

In the simulation, the flow field consists of conductive liquid and insulating fluid (water and water vapor are used in this paper). According to the EWOD principle, an electric field is generated by applying a voltage to the solid surface, which changes the equilibrium state of forces. It can adjust the contact angle between the droplet and the wall surface.

The effect of voltage on the contact angle variation was simulated to verify the correctness of the LB-ED method. In this paper, the computational area is set as a finite mesh space of 600 × 150. The left and right boundaries are solid wall surfaces with no-slip half-way bounce boundary conditions. The upper and lower boundaries are applied to periodic boundary conditions. The chemical potential multiphase model can effectively simulate the wetting phenomenon by setting a constant chemical potential $\mu_{s}$ on the solid surface to mimic substrates with different hydrophobicity. The density distribution of the flow field is initialized by [58]:

(32) $$\rho \left(x,y\right)=\frac{\rho_{g}+\rho_{l}}{2}+\frac{\rho_{g}-\rho_{l}}{2}\tanh\left[\frac{2\left(D-D_{0}\right)}{w}\right],$$

where $\rho_{g}$ and $\rho_{l}$ are two-phase coexistence densities, which can be obtained by Maxwell’s equal area method at $T_{r}=0.6$, ${D_{0}}^{\prime }=60$ is the thickness of the initial lens, and *w* is the width of the gas–liquid transition zone under the lattice unit. In the simulation, mirror diameter is L=1.5 mm, gas–liquid surface tension $\gamma_{lg}=0.072$ N/m, superlarge density ratios coefficient k=0.2, and the relaxation time is taken as τ=0.8. We compare the experimental results of numerical simulation with the theoretical results of the Lippmann–Young equation, and the model is in good agreement with the theoretical results, which confirms the correctness of our model. Firstly, the flow field is initialized on a substrate with a voltage of 0 V using Equation (35). Due to the hydrophobicity of the substrate, the droplets will evolve and eventually form a stable biconvex lens on the substrate. Secondly, every $5\times 10^{4}$ time steps, the voltage is increased by 0.05 V to simulate the change of contact angle at different voltages. The following equation can be used to calculate the dynamic contact angle:

(33) $$\cos\theta =-\frac{R}{r},$$

where *R* is the lens barrel radius, and *r* is the lens’s curvature radius. *r* can be calculated from analytic geometry:

(34) $$r=\frac{m^{2}+R^{2}}{2m},$$

where *m* is the vertical distance from the midpoint of the lens surface to the chord of the two three-phase contact points on the arc. When the lens is concave, m<0.

**Figure 3:**
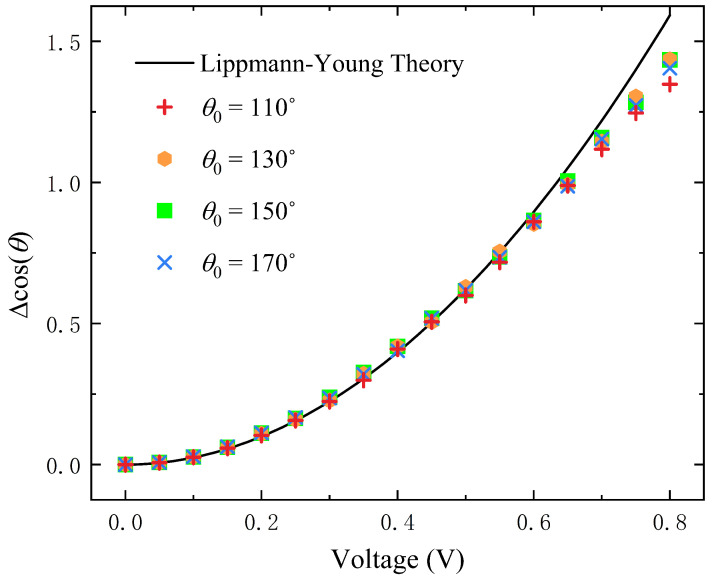
Contact angle variation with voltage on different hydrophilic substrates and compared with Lippmann–Young’s analytical solution.

As shown in Figure 3, when the initial contact angles are 110°, 130°, 150°, and 170°, the change of the contact angle increases nonlinearly with the increase of voltage. The results are consistent with the Lippmann–Young equation at low voltage. These also verified that wall wettability does not change the effect of voltage on the lens. Since the electric field has an electrostatic traction effect on the gas–liquid interface near the three-phase contact point, a combined force parallel to the solid wall direction is generated, which leads to the deformation of the lens and the decrease of the macroscopic contact angle. When the voltage exceeds a certain value, the simulation results deviate from the Lippmann–Young equation, and contact angle saturation occurs. Vallet et al. [24,59] also observed contact angle saturation in dielectric wetting experiments, justifying the LB-ED method in this paper.

## 4. Results and Discussion

### 4.1. The Effect of Voltage on the Focal Length of the Lens

The relationship between the focal length of the lens and the radius of curvature is

(35) $$\frac{1}{f}=\left(n_{2}-n_{1}\right)\left[\frac{1}{r_{1}}-\frac{1}{r_{2}}+\frac{\left(n_{2}-n_{1}\right)h}{n_{1}n_{2}r_{1}r_{2}}\right],$$

where $r_{1}$ and $r_{2}$ are vector quantities that represent the radii of curvature of the two refractive surfaces, $n_{1}$ and $n_{2}$ are the refractive index of conductive liquid and insulating gas, respectively, and *h* is the lens’s thickness. In this paper, Lensmaker’s formula is used, and the last term in Equation (35) can be removed, $r_{1}=-r_{2}$. Bringing Equation (34) into Equation (35), it is easy to obtain the focal length formula:

(36) $$f=\frac{m^{2}+R^{2}}{4m(n_{2}-n_{1})}.$$

For a 589 nm light source, the refractive index of air and water is $n_{1}=1.0$ and $n_{2}=1.333$ respectively. In this simulation, the relaxation time is taken as τ=0.8. The theoretical relationship between the voltage and the focal length of the liquid lens can be obtained through some simple operations of Equations (6), (33) and (35):

(37) $$f=-\frac{R}{2\left(n_{2}-n_{1}\right)\left(\cos\theta_{0}+\frac{\varepsilon_{0}\varepsilon_{r}}{2\gamma d}v^{2}\right)}.$$

**Figure 4:**
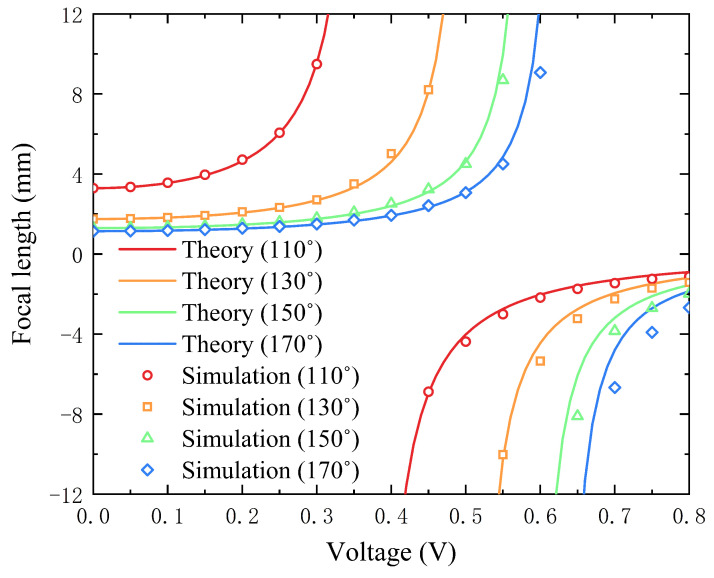
The numerical focal length on different wettability substrates and voltages compared with the theoretical focal length in initial contact angles 110°, 130°, 150°, and 170°.

Since the substrate is hydrophobic, it can be seen from Figure 4 that the initial focal length of the liquid lens is positive. Applying a voltage can change the wettability of the substrate. In the first stage, the lens’s focal length grows slowly and then quickly, and the lens has a convex shape. The focal length changes slowly at the initial stage. Due to the confinement effect of the hydrophobic substrate on the conducting liquid, the effect of the electric field force on the contact angle appears negligible at low voltages. Berge et al. [20]. Experimental measurements found that the lens’s focal length does not change significantly when the voltage is below a certain value. The lens’s focal length varies faster when the voltage reaches a certain value. The simulation results in this paper agree with the experimental results [60]. When the voltage increases to a certain point, the shape of the liquid interface is close to flat, and the lens’s focal length is infinite at this point. Then, the lens changes from convex to concave, and the focal length varies from positive to negative infinity. As the voltage increases, the focal length rises rapidly from negative until it reaches a relatively stable negative value [61]. The reason is that the contact angle between the conductive liquid and the inner wall of the lens cavity reaches saturation at high voltage.

The refractive index of light of different wavelengths on the same optical medium is different, so the imaging of light of different wavelengths through the medium will produce chromatic aberration. The combination of solid and liquid is used to realize apochromatic aberration to improve image quality.

### 4.2. The Effect of Liquid Viscosity on the Performance of the Lens

**Figure 5:**
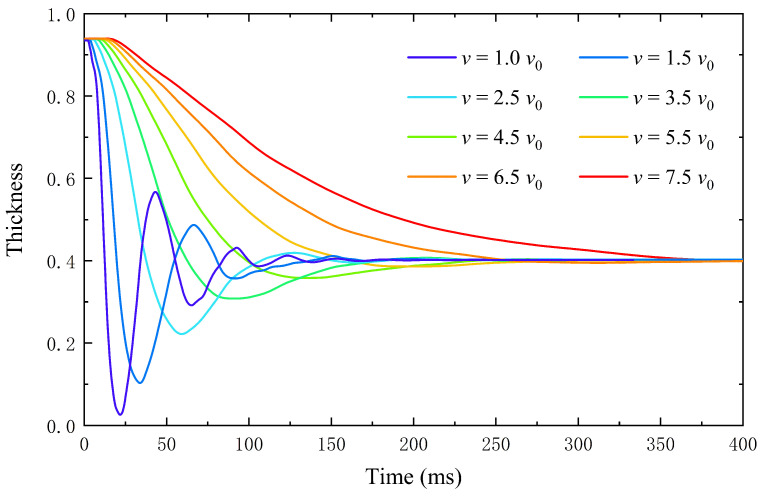
The thickness of the center of the lens changes with time under different viscosity conditions.

In this section, we simulate the dynamic process of the lens during zooming. Figure 5 shows the variation of the lens center thickness with time for different viscosity liquid conditions. After the voltage is applied, the thickness of the lens changes slowly at first, then decreases rapidly, and finally reaches a stable state after a period of oscillation. This is because the electric field force causes the contact angle to change abruptly at the instant of applying voltage. Due to the center point being far from the substrate, the lens center needs a delay time to respond to the change in contact angle so that no thickness change is detected in the initial stage.

The dynamic process of zooming is simulated under different viscosity *v* conditions to analyze the lens stability factors. When τ=0.7, $v=v_{0}$. From Figure 5, it is found that, when $v<5.5v_{0}$, the zooming process of the lens is in the under-damped state, and it takes several oscillations to reach the steady state; when $v=5.5v_{0}$, the system is close to the critical damped state; when $v>5.5v_{0}$, the system is in the over-damped state, and it takes longer for the lens to enter the steady state; when the system is in the near-damped state, the lens takes the shortest time to reach the steady state, and the system performance is the best. When the voltage is the same, the thickness of the midpoint of the lens eventually remains at the same level. That is, viscosity has no effect on the change of interface position but significantly affects the system response time and stability. Therefore, to a certain extent, the liquid with appropriate viscosity can give the system a satisfactory response speed.

### 4.3. Accelerate Lens Zoom with an Overdrive Voltage

When the liquid viscosity coefficient of the electrowetting lens used in the simulation is large, the liquid exhibits an overdamped state. The main reason for the long zooming time is the slow speed of liquid motion. Applying the overdrive voltage for a short time can effectively accelerate the movement of the liquid surface and reduce the zoom time. However, if the overdrive time is too long, the liquid surface will cross the equilibrium position due to inertia, which is not conducive to reducing zoom time [62]. In this case, the zoom time can only be slightly reduced or even increased. If the overvoltage time is too short, the function of the overvoltage drive cannot be fully exerted. The lens zoom speed is not only related to the duration of overvoltage but also the degree of overvoltage. The degree of overvoltage should not be too large or small. If the degree of overvoltage is too small, the interface deformation speed is not fast enough, and the effect of accelerated zooming is not apparent; if the degree of overvoltage is too large, the system load may be exceeded.

**Figure 6:**
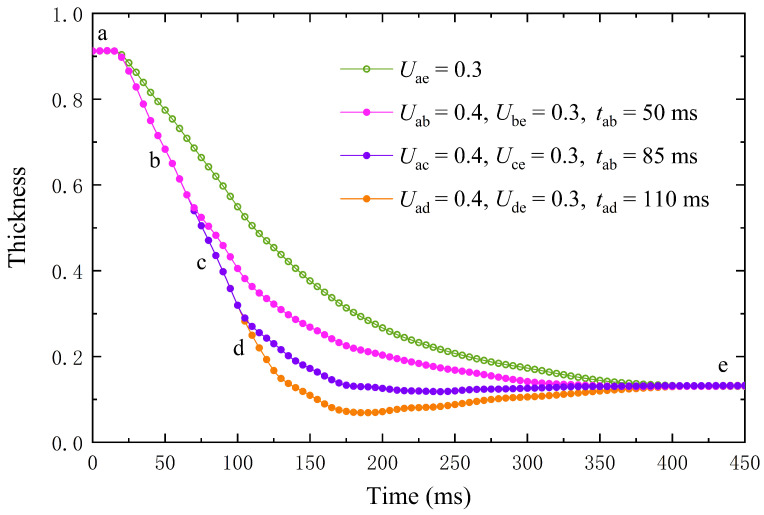
Variations of lens center height during applied voltage for high viscosity liquid lens. Take points b, c, and d as dividing lines, the left side is overvoltage, and the right side is target voltage. Point a is the initial state, and point e is the final steady state after applying the target voltage.

This simulation assumes that the maximum voltage the device can withstand is 0.4 V. The liquid viscosity coefficient is $v=7.5v_{0}$. The overvoltage duration of 0.4 V is used for 0 ms, 50 ms, 85 ms, and 110 ms. Then, it switches to the target voltage of 0.3 V. As shown in Figure 6, the deformation speed under overvoltage is significantly accelerated. In the initial stage, the thickness of the lens center hardly changes. The distance between the lens center and the substrate is long, and it takes some time for the middle of the lens to respond to the voltage change. Subsequently, the lens interface rapidly approaches the equilibrium position under the overvoltage condition. After the conversion to the target voltage, the lens center position continues to approach equilibrium until it stabilizes. Under the different overvoltage time, the zoom time is 380 ms, 330 ms, 200 ms, and 380 ms, respectively. The zoom works best when overvoltage is applied for 85 ms. The lens realizes motion acceleration, and the interface does not exceed the stable position. If the overvoltage time continues to increase, the interface will approach the equilibrium position more quickly. However, due to inertia, the interface rushes past the equilibrium position and the zoom time rises instead. It can be seen that the overvoltage voltage can reduce the zoom time. However, too long overvoltage time is not conducive to lowering zoom time. In the best case, the zoom time can be reduced from 380 ms to 200 ms.

### 4.4. Acceleration Lens Zoom Is Driven by Undervoltage

When the viscosity coefficient of the liquid used in the experiment is small, the liquid exhibits an underdamped state. The main reason for lens oscillation is that inertia causes the interface to continue moving after reaching its target position. Under the combined effect of solid–liquid interfacial tension, solid–gas interfacial tension, gas–liquid interfacial tension, and electric field force, the amplitude of interfacial oscillation gradually decreases. When the four forces reach equilibrium, the interface reaches a stable state.

Minimizing the effect of inertia on the lens can effectively reduce the zoom time. To reduce zoom time, drive the lens with undervoltage. When the interface moves near the equilibrium position, switch back to the target voltage [62]. At this time, the inertial force is the lowest, and choosing this moment as the voltage switching point can effectively reduce oscillations. If the overvoltage time is too long or too short, the interface will oscillate greatly, which is not conducive to lowering the zoom time. In this case, interface stabilization can only be accelerated slightly, and accelerated zooming has a limited effect.

**Figure 7:**
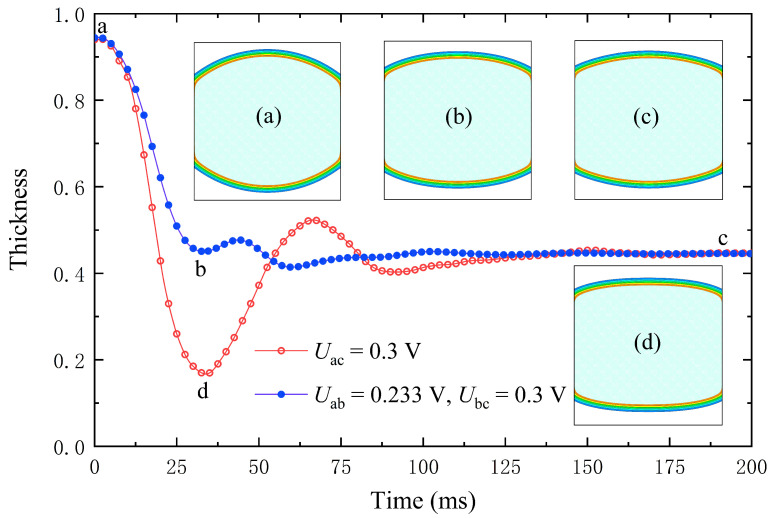
Variations of lens center height with time during applied voltage in a low viscosity liquid lens. The blue line depicts the modified zoom method, where a low voltage is applied and then switched to the target voltage at point b. Insets (a–c) show the lens’s state diagram at different times at two voltages. Inset (d) shows the state diagram at 35 ms under a single voltage.

In this simulation, $v=1.5v_{0}$, an undervoltage of 0.233 V is used for 35 ms and then switched to the target voltage of 0.3 V. From insets (a) and (c) in Figure 7, the curvature of the lens interface is changed by the electric field force, when a voltage is applied to the substrate. From insets (b)–(d) in Figure 7, the stable position can be reached faster using two voltages. After switching to the target voltage, a small oscillation occurs in the center of the lens. Using undervoltage driving can effectively shorten the zoom time from 160 ms to 100 ms.

### 4.5. Influence of Driving Voltage on the Moving Speed of the Lens

In order to understand the influence of drive voltage on the moving speed of the lens, we conducted a simulation of liquid lens movement zoom. The electrode array is selectively opened to cover approximately one-third of the solid–liquid interface. Since the voltage is only applied to part of the substrate, the lens potential is asymmetrically distributed. The advancing side has a higher potential than the receding side, with a larger potential gradient, resulting in a resultant force for forwarding motion. Applying the proper electrode switching frequency, the lens will move quickly and smoothly [63,64].

**Figure 8:**
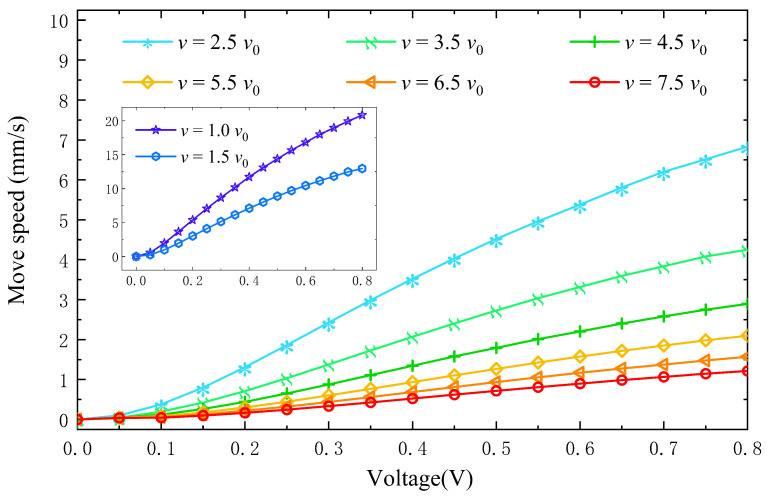
Variation of lens movement speed with voltage for different viscosity liquids.

The voltage in this experiment was changed from 0 V to 0.8 V in increments of 0.05 V each time. Figure 8 shows the variation of the lens movement velocity with time for different voltage and viscosity conditions. The movement of the lens increases nonlinearly with increasing voltage at different viscosities. At the same viscosity factor, the lens can obtain faster motion at higher voltages. The chosen liquid viscosity also dramatically impacts the speed of lens movement. The speed of lens movement decreases with increasing viscosity, which means that higher viscosity requires higher voltage to overcome the resistance. From the simulation, it can be concluded that the position and shape of the lens can be accurately adjusted by simply changing the voltage. The feasibility of the lens shift and focus adjustment method is demonstrated.

## 5. Conclusions and Outlook

In this paper, we proposed a movable and focus-tunable EWOD liquid lens. The dynamic evolution of the lens zoom was analyzed using the LB-ED method. The effect of voltage on the focal length of the lens was studied. Through the simulation of the liquid lens with different viscosities, it can be found that viscosity greatly influences the zooming time. The lens shows a damped state at high viscosity and an oscillating state at low viscosity. At the initial moment of applied voltage, the contact angle changes abruptly under electric field force and tension combined. However, the lens needs time to respond to the change in contact angle. Additionally, methods were proposed to accelerate lens stabilization at low and high viscosities, achieving speed improvements of about 30% and 50%, respectively. Simulations of lens motion at different viscosities demonstrate that higher viscosity liquids require higher voltages to achieve the same movement speed. The results of this study contribute to the design and analysis of adaptive zoom lens. Three-dimensional analysis and simulations concerning EWOD lens will be performed in future work.

## Data Availability

Not applicable.
